# Evaluation of Fasting and Probiotics in Reducing Postweaning Stress in Rabbits: Study of their Effects on Biochemical and Gene expression Patterns

**DOI:** 10.1007/s12010-023-04479-w

**Published:** 2023-05-09

**Authors:** Fatma Abou-Hashim, Walaa H. Khalifa, Mohamed B. Shalaby, Salwa M. Kassem, Wagdy K. B. Khalil

**Affiliations:** 1 Animal Production Department, Faculty of Agriculture, Cairo University, Giza, Egypt; 2 Animal Production Department, Agricultural and Biological Researches Institute, National Research Centre, Dokki, Giza, Egypt; 3 Toxicology Department, General Organization of Teaching Hospitals and Institutes, Research Institute of Medical Entomology, Ministry of Health and Population, Giza, Egypt; 4 Department of Cell Biology, Biotechnology Research Institute, National Research Centre, Dokki, Giza, Egypt

**Keywords:** Postweaning stress, Gene expression, DNA damage, Probiotics, Fast, Hormonal disturbance

## Abstract

Postweaning stress in mammalian in vivo models leads to significant oxidative stress in the body as well as inducing hormonal disturbance. In this study, we assessed progressive alterations in reactive oxygen species (ROS), which at high levels can show oxidative stress, in addition to oxidative damage to the DNA structure of rabbits. Different groups of rabbits were fasted for 48 h per week for 3 weeks, fed a commercial diet with probiotics added (200 mg of *Bacillus licheniformis* and *Bacillus subtilis*), and fasted while being treated with probiotics. The results showed that weaning induced a significant elevation in oxidative stress markers, such as the ROS-related genes malate dehydrogenase 1 (MDH1) and flavin-containing monooxygenase 2 (FMO2), DNA damage, and hormonal disturbance. However, probiotic treatment resulted in significant decreases in the levels of malondialdehyde, cortisol, and triiodothyronine (T3); DNA damage; and apoptosis, as well as changes in the expression of ROS-related genes. On the other hand, supplementation with probiotics reduced these postweaning stress signs in fasted animal models by elevating the genes encoding catalase and superoxide dismutase as well as increasing glutathione peroxidase (GSH-Px), glutathione–s-transferase, alkaline phosphatase, glucose, and thyroxin (T4) levels. The results suggest that supplementation with probiotics accompanied by a fasting program could decrease oxidative stress, ROS genes, and genomic DNA damage and improve the hormonal status that is induced by postweaning stress in mammalian in vivo models.

## Introduction

The digestive process of the rabbit is highly sophisticated and sensitive. This explains the rabbit’s sensitivity to enteric disorders, especially with exposure to stress. During aning, the diet of the rabbit is changed from milk to solid food. This sudden change can increase the rabbit’s vulnerability to digestive tract problems and microbial diseases [1]. Moreover, for the young mammal, the weaning and postweaning periods are particularly important for growth and feeding efficiency [2, 3]. Fasting in growing rabbits can have advantages, such as increased digestive efficiency, modification of the partition of body energy retention to protein instead of fat, and reduction of mortality and morbidity due to digestive problems [4]. Moreover, the amount and duration of restricted feeding is involved in the regulatory mechanisms of metabolism in animals. Restricted feeding may positively affect several changes in metabolic disorders that lead to hormonal changes, immune depression, and altered digestive system functions, especially in the liver and small intestine [3]. Furthermore, fasting can quickly retrieve the morphology and functions of the intestine [5]. Increase in the endogenous production of adrenal corticoids from the adrenal glands following the action of stress factors such as fasting has been demonstrated [6]. On the other hand, a long period of nutrient reduction during development results in homeostatic reactions of the corticotropic, somatotropic, and thyrotropic axes [7]. In addition, weaning leads to significant oxidative stress and apoptosis in the body as well as inducing reactive oxygen species (ROS)-related genes and hormonal disturbances [8, 9]. Preliminary evidence suggests that probiotics have a potent therapeutic advantage in this condition [10, 11].

It is known that DNA is the main target of oxidative stress on mammalian cells. Many studies have shown that the pathophysiology of many diseases, such as diabetes, atherosclerosis, cancer, and neurological diseases, is caused by persistent oxidative damage to DNA [12]. Oxidative damage to the genetic materials in the genome depends mainly on the susceptibility to oxidative attack on the nucleotide sequences of the genetic material, and the ability for successful repair processes. Studies have shown that one of the most vulnerable DNA nucleotide sequences to attack and oxidative damage are the non-coding sequences due to its basic structure [13]. It was found that the most nitrogenous bases of DNA for oxidative modification are guanine compared to the other three nitrogenous bases on the genetic material. As a result of the oxidative attack of nitrogenous bases, 8-Hydroxyguanine (8-OH-Gua) is formed as one of the common marker of oxidant-induced DNA damage [14].

Probiotics are living microorganisms like; yeast, active bacteria, or bacterial spores’ possess the ability to prevent enteric diseases in rabbits. Probiotics encourage gut colonization as well as stabilizing eubiosis by competing with the growth of harmful microorganisms and enhancing digestive enzymes and vitamin secretion by lowering the intestinal pH. Probiotic preparations are added to the diet to enhance fermentation in the digestive system. Probiotics include *Bacillus licheniformis* and *Bacillus subtilis*. Probiotic spores have the ability to persist in the bacterial pelletization process [15] and passage through the stomach. Probiotic spores can sprout in the rabbit’s intestines and consume carbohydrates in huge amounts for their growth, in addition to manufacturing digestive enzymes such as amylase, protease, and lipase. These enzymes catalyze and improve the digestion of food. During weaning, the digestive system passes through an adaptation stage. In this stressful state, small rabbits are more sensitive to illnesses, and they grow slowly [16]. Therefore, administration of probiotics accompanied by a fasting regime could relieve weaning shock and alleviate oxidative stress in addition to reducing mortality and morbidity. We tested this hypothesis on recently weaned male white New Zealand rabbits.

## Materials and Methods

### Animal Management and Feeding

This study was performed at a rabbit farm of Animal physiology Lab, Faculty of Agriculture, Cairo University, Giza, Egypt. Twenty four recently weaned 5-week-old male white New Zealand rabbits (525 ± 8.34 g. BWT) were randomly distributed into four experimental groups (n = 6). The rabbits were housed in a naturally ventilated building and kept in individual wire galvanized cages. The rabbits were fed according to NRC [17] allowances on a concentrated feed mixture (crude protein 20%, crude fat 3%–4%, crude fiber 14%–15%, net energy 2640 kcal) mixed with Bersem Hegazy hay as roughage. Animals were offered their CFM once daily to measure feed consumption (FC) and feed conversion rate (FCR). The ingredients of the pellets are shown in Table 1.Water was available ad libitum, and the lighting program provided 16 h light and 8 h dark per day. This study was approved by the Institutional Animal Care and Use committee of Cairo University, protocol No. (CU-II-F37-17).

**Table 1:** Ingredients of the feed

Ingredients name	
Yellow corn	Anticoccidia
Wheat bran	D.L methionine
Soybean meal	Lysine
Barley	Limestone (CaCO_3_)
Dry yeast	Salt (NaCl)
Antifungal	Mineral premix*

^*^1 kg of feed contains 10.000 mg of copper, 800 mg of iodine, 150 mg of selenium, 340.000 mg of calcium, and 200 mg each of iron, manganese, zinc, and cobalt

### Experimental Design

The rabbits were allocated to four groups. Group 1: The rabbits were fed commercial diet ad libitum and served as a control group (Ad). Group 2: The rabbits fed the commercial diet ad libitum were fasted for 12 h/day twice a week (F12) for 3 weeks. Group 3: The rabbits were fed the commercial diet with 200 mg of prebiotics (*Bacillus licheniformis* and *Bacillus subtilis*) per liter of water for 3 weeks, (biofactor liquid). Group 4: The rabbits fed commercial diet were fasted for 48 h each week (F48) with 200 mg of prebiotic (*Bacillus licheniformis* and *Bacillus subtilis*) per liter of water for 3 weeks. During the last week of the experiment, all groups were fed ad libitum.

### Growth Performance Traits

The animals’ body weight, daily feed intake, and the number of dead rabbits were recorded. The daily animal weight gain with its FCR was recorded per week, in addition to daily recording of the mortality rate during the experimental period (8 weeks).

### Carcass Characteristics

By the end of the study, four rabbits (8 weeks of age) in each treatment group were chosen randomly, fasted for 12 h, weighed, and sacrificed. Then, the kidney, spleen, liver, and cecum were taken for molecular biological analysis.

### Blood Samples for Determination of Oxidative Status Biomarkers

After the last day of experimental treatment, the animals were fasted overnight, and blood samples were collected. Blood plasma samples were separated into lithium heparin test tubes and centrifuged at 4000 rpm for 15 min; the plasma was then stored in a deep freezer at approximately − 20ºC until analysis. Total antioxidant capacity (TAC), total superoxide dismutase (T-SOD), catalase (CAT), and malondialdehyde (MDA) were analyzed as described by Koracevic et al. [18] and Richard et al. [19]. In addition, blood glutathione peroxidase (GPx) and glutathione–s-transferase (GST) activities were assessed according to Miranda et al*.* [20].

### Hormone Assay

Blood plasma concentrations of thyroxin (T4) and triiodothyronine (T3) were measured according to Abdel-Fattah et al. [21] using the radioimmunoassay (RIA) technique. Blood plasma concentrations of cortisol were evaluated by RIA, using the CORT kit (ICN Biomedical Inc., Costa Mesa, CA, USA) according to Palme et al. [22].

### Comet Assay

Comets were examined in liver samples according to Blasiak et al. [23]. Low-melting-point agarose was mixed with liver specimen (1:10 v/v). The mixture of the samples was loaded onto slides which were coated previously with normal agarose. The slides were kept in a dark place followed by covering it with low-melting-point agarose and left for 30 min at 4 °C. The slides were conserved in lysis solution for one hour followed by covering it in alkaline unwinding solution for another 1 h. After electrophoresis run the slides were washed in neutralizing buffer followed by immersing it in 70% ethanol. An ethidium bromide dye was used to stain the slides which were finally examined by a Zeiss epifluorescence microscope [24, 25].

### Expression of Antioxidant- and ROS-Related Genes

#### Total RNA Isolation

Liver specimens were used to insulate the RNA of the rabbit’s tissues by TRIzol® Reagent (Invitrogen, Germany). The RNA molecules were confirmed in agarose gel (1.5%) stained with ethidium bromide to verify of the presence 28S and 18S bands. After assessment the purity of the RNA aliquots of the ribonucleic acids were prepared [26] for reverse transcription (RT).

#### RT Reaction

Segregated RNA from rabbit liver specimens was utilized to obtain cDNA through reverse transcription reaction using RevertAidTM First Strand cDNA Kit (Fermentas, Germany) containing oligo(dT)18 primer, dNTP Mix, Reverse Transcriptase enzyme and RNase Inhibitor. The reaction PCR products were used immediately for qRT-PCR or kept at − 20ºC up to use.

#### qRT-PCR Analysis

StepOne™ Real-Time PCR System (Applied Biosystems, USA) was used for amplification of the obtained cDNA using 1 × SYBR® Premix Ex TaqTM (TaKaRa, Biotech. Co. Ltd, China). The primer sequences of the specific genes tested (CAT, SOD1, MDH1, FMO2) designed using (Primer3 software) are presented in Table 2 [27]. The 2^*−*ΔΔCT^ method was used to determine the quantity of the target.

**Table 2:** Primer sequences

Gene	Primer sequence	NCBI reference
CAT	F: CTG AAC ATC ATC ACG GCA GG	XM_002709045.3
	R: CAC CTT CGC CTT GCT GTA TC	
SOD1	F: ACC TGG GTA ATG TGA CTG CA	NM_001082627.2
	R: CAA TGA CAC CAC AGG CCA AA	
MDH1	F: GAT GAC AGC TGG CTC AAA GG	XM_002709844.3
	R: GCC ATC AGA GAT GAC ACC CA	
FMO2	F: AAC TGA TCC GTT GAC CAC CT	XM_017345488.1
	R: ATT GGG TCT GCA GGA AAG GA	
β-Actin	F: ACC TGA CCG ACT ACC TCA TG	AF309819.1
	R: CTC GTA GCT CTT CTC CAG GG	

*CAT*, catalase; *SOD1*, superoxide dismutase 1; *MDH1*, malate dehydrogenase 1; *FMO2*, flavin-containing monooxygenase 2

### Apoptosis Assay

Liver tissue samples were homogenized into single-cell suspensions by the method of Khalil et al. [28]. We detected apoptosis of cells by flow cytometry (FCM) assay using an Annexin V/propidium iodide (PI) apoptosis detection kit. The single-cell suspension (1 × 106 cells/mL) was put into 200 μL of ice-cold binding buffer, and 10 μL of horseradish peroxidase FITC-labeled Annexin V and 5 μL of PI was added. The cell suspension was incubated for 15 min in darkness at room temperature. The apoptosis rate was detected by flow cytometer as normal cells (FITC and PI-negative cells) and apoptotic or necrotic cells (FITC and PI-positive cells).

### Statistical Analysis

The data were analyzed by the General Linear Models technique of the Statistical Analysis System (SAS, 1982), then by the Scheffé test to evaluate significant differences between groups. The results are presented as means ± SEM. All significance statements were based on P < 0.05.

## Results and Discussion

The motive of this study was to assess the protective impact of probiotics administration accompanied by a fasting regime against postweaning oxidative stress, DNA damage, and hormonal disturbances for reducing mortality and morbidity in recently weaned male White New Zealand rabbits.

### Growth Performance Traits

As shown in Table 3, there were no significant effects of fasting regimen and probiotics on final body weight and total body weight gain of growing rabbits. The study results agreed with those of El-Speiy et al. [29] and Beshara et al. [30], who repoted that probiotics did not influence rabbits’ daily weight gain in comparison with control animals. Mancini and Paci [16] reported that feeding restriction in growing rabbits (5–12 weeks) had no marked effect on body weight or daily weight gain. However, fasting of growing rabbits, with or without the addition of probiotics for 8 weeks postweaning, caused highly significant decreases in feeding intake by 38.5% and 33%, respectively, compared with the control group. The present results agreed with those of Sherif et al. [31], who found an extremely significant improvement in feed intake in the growing rabbits group with the ad libitum ones (control group) as compared with other feed restriction groups through all study intervals (5–13 weeks of age), and the FCR was highly significantly increased in growing rabbits with feed restriction system (FRS) (90% or 80%) in comparison with ad libitum animals during all periods of the experiment. No deaths occurred during the experimental period; these results agree with several previous studies which showed that restricted feeding did not affect the mortality rate in weaning rabbits [30, 32–34]. Other studies reported that long periods of restricted feeding (2 to 3 weeks) in growing rabbits decreased mortality and morbidity from digestive problems [2, 24]. Furthermore, previous studies documented that the viability of rabbits was improved from 2 to 16% when probiotics were added to their diet [21, 30].

**Table 3:** The rabbit’s growth performance and the mortality rates

Measurement	Control	Fasted (starvation)	Probiotics	Fasted + probiotics
Initial BW (gm)	904.40 ± 102.01	883.60 ± 65.107	891.20 ± 52.15	906.00 ± 71.72726
Final BW (gm)	1751.20 ± 85.469	1769.60 ± 72.543	2097.60 ± 72.22	1919.20 ± 88.85
Total feed intake (gm)	3572.90 ± 54.99^b^	2199.4 ± 35.14^c^	3900.0 ± 104.88^a^	2382.6 ± 47.95^c^
BWG (gm)	846.80 ± 103.3	886.00 ± 81.05	1206.4 ± 104.02	837.20 ± 190.82
FCR	4.47 ± 0.54^a^	2.55 ± 0.19^b^	3.33 ± 0.31^b^	2.34 ± 0.17^b^
Mortality	Nil	Nil	Nil	Nil

Data are shown as means ± SEM. Mean values within weights with different superscript letters are significantly different (P < 0.05)

### Biomarkers of Serum Oxidative Status

The effects of fasting, probiotic supplementation, and fasting with probiotic supplementation on serum MDA, SOD, CAT, and TAC are shown in Table 4. Notably, growing rabbits during the fasting period had significantly elevated concentrations of serum MDA, an indicator of lipid peroxidation, by about 31% compared with rabbits in the control group; however, the lowest values were recorded in the ad libitum fed group in the presence of probiotic supplementation. This result may be due to severe nutrient deprivation, which causes increased oxidative stress as a result of the excessive production of ROS in mitochondria during cellular respiration; liver membranes are more sensitive to oxidative damage [35]. Nurmasitoh et al. [34], Kleniewska et al. [35], as well as Ayyanna and Ankaiah  [36] reported that the lower percentage of fat in growing rabbits during fasting is probably one of the factors causing the increase in plasma MDA levels. However, serum SOD levels were significantly higher in the probiotic supplementation group (1143.6.0 mM/L) compared with other fasted groups with or without the addition of probiotics (1039.0 and 598.42 mM/L, respectively). The probiotic group had a slightly increased catalase level (CAT) and plasma TAC compared with other groups. These results may be due to reduced output of toxins or antimicrobial substances by other microorganisms, competition for adhesion to epithelial cells, increased resistance to colonization, prompting of the host’s immune system, and reduced stress in rabbits [16, 37].

**Table 4:** Oxidative status biomarkers

Marker	Treatment group			
	Control	Fasted	Fasted + probiotics	Probiotics
MDA (nmol/ml)	307.84 ± 81.76^ab^	443.05 ± 34.63^a^	403.15 ± 67.88^ab^	253.41 ± 23.60^b^
CAT (U/L)	271.58 ± 6.78^a^	265..53 ± 5.18^a^	276.63 ± 12.11^a^	284.21 ± 4.28^a^
SOD (mM/L)	948.50 ± 10.32^b^	598.42 ± 68.34^c^	1039.0 ± 19.67^ab^	1143.6.0 ± 4.96^a^
TAC (mM/L)	0.55 ± 0.05^a^	0.51 ± 0.04^a^	0.54 ± 0.063^a^	0.72 ± 0.14^a^

Mean values within tissues with different superscript letters (^a, b^) are significantly different (P < 0.05)

### Determination of Activity of Glutathione Peroxidase, Glutathione-S-Transferase, and Alkaline Phosphatase

Table 5 shows the activity levels of the antioxidant enzymes glutathione peroxidase (GPx) and glutathione-S-transferase (GST) in normal and fasted rabbits fed probiotics. The activity levels of GPx and GST in blood samples of fasted rabbits were reduced (5.3 ± 0.04 U/mg tissues/min and 2.1 ± 0.02 µmol/mg protein, respectively) compared with those in control rabbits (6.7 ± 0.07 U/mg tissues/min and 2.7 ± 0.03 µmol/mg protein, respectively). However, the GPx and GST activities were significantly (P < 0.05) increased (8.4 ± 0.11 U/mg tissues/min and 3.5 ± 0.06 µmol/mg protein, respectively) in rabbits supplemented with probiotics compared with fasted rabbits (5.3 ± 0.04 U/mg tissues/min and 2.1 ± 0.02 µmol/mg protein, respectively). Moreover, the activity levels of GPx and GST were increased (7.3 ± 0.03 U/mg tissues/min and 2.6 ± 0.04 µmol/mg protein, respectively) but without significant differences in fasted rabbits supplemented with probiotics compared with fasted rabbits (5.3 ± 0.04 U/mg tissues/min and 2.1 ± 0.02 µmol/mg protein, respectively). The probiotics have potential antioxidant effects [38, 39]. The ALP activities have no significant difference in all study groups in comparing with its level in control group.

**Table 5:** Glutathione peroxidase, glutathione-S-transferase, and alkaline phosphatase (ALP) activities in blood samples of control and fasted rabbits fed standard diet with probiotics

Treatment	Glutathione peroxidase activity (U/mg tissues/min)	Glutathione-S-transferase activity (µmol/mg protein)	ALP activity (mU/mL)
Control	6.7 ± 0.07^b^	2.7 ± 0.03^ab^	134.86 ± 3.12
Fasted (starvation)	5.3 ± 0.04^b^	2.1 ± 0.02^b^	119.35 ± 23.61
Probiotics	8.4 ± 0.11^a^	3.5 ± 0.06^a^	122.46 ± 4.43
Fasted + probiotics	7.3 ± 0.03^ab^	2.6 ± 0.04^ab^	120.17 ± 3.01

Means with different superscripts ^(^^a, b)^ between groups in the same treatment week are significantly different (P < 0.05)

### Thyroid Hormones, Cortisol, and Glucose Levels

Triiodothyronine (T_3_) and thyroxin (T_4_) levels are shown in Table 6. The results display that there were no significant effects of the fasting regime and probiotics on serum T_3_ concentrations. However, plasma T_4_ concentrations were increased in the probiotic group with or without a fasting period when compared with those in the control and fasting groups [38]. This result may be due to feeding restrictions having a role in the regulatory mechanisms of metabolism in animals. Regarding plasma glucose and cortisol hormones (Table 6), the results did not show any significant differences (P < 0.01) between any experimental groups. However, there was a very small, nonsignificant decrease in cortisol levels in the fasted groups; this result was in agreement with Van Harten and Cardoso [39], who observed that blood glucose was not affected by restricted feeding in NZW rabbits. On the other hand, Rajman et al. [40] found a decrease in plasma glucose with a short (24 h) period of food restriction in chickens. Similarly, Jamshed et al. [41] and Grigorova et al. [42] observed that a slight restriction (50 g feed/head/day) reduced blood glucose during the feed restriction period.

**Table 6:** Thyroid hormones, cortisol, and glucose levels

Parameter	Treatment group			
	Control	Fasted (starvation)	Probiotics	Fasted + probiotics
T3 (ng/dl)	3.06 ± 0.42^a^	2.41 ± 0.38^a^	2.500 ± 0.26^a^	2.26 ± 0.29^a^
T4 (ug/dl)	51.66 ± 10.14^b^	60.51 ± 5.67^b^	80.95 ± 3.00 ^a^	82.45 ± 5.53^a^
Cortisol (ug/dl)	103.99 ± 5.68^a^	108.09 ± 2.89^a^	105.94 ± 3.60^a^	100.00 ± 1.622^a^
Glucose (mg/dl)	134.81 ± 3.08^a^	130.74 ± 4.39^a^	136.64 ± 3.24^a^	144.84 ± 1.33^a^

Mean values within tissues with different superscript letters (^a, b^) are significantly different (P < 0.05)

### Determination of DNA Damage

Table 7 shows the effect of probiotics feeding against starvation stress-induced DNA damage in male rabbits. The rate of DNA damage in fasted rabbits was increased (9.82 ± 0.86) significantly (P < 0.05) compared with control rabbits (6.80 ± 0.58). In contrast, supplementation of control or fasted rabbits with probiotics significantly decreased (P < 0.05) the rates of DNA damage (6.23 ± 0.80 or 7.41 ± 0.93, respectively) compared with those in fasted rabbits (9.82 ± 0.86). Ebeid et al. [43] and Uhlirova et al. [44] explained the positive protective effect of probiotics on DNA due to prevention of oxidative DNA damage and cellular oxidation as well as an antigenotoxicity effect.

**Table 7:** Scores of DNA damage in liver tissues of control and fasted rabbits fed standard diet with probiotics

Treatment	No. of samples	No. of cells		Class^**^				DNA-damaged cells (%)
		Analyzed^*^	Comets	0	1	2	3	
Control	5	500	34	466	29	5	0	6.80 ± 0.58^b^
Fasted (starvation)	5	500	49	451	30	13	6	9.82 ± 0.86^a^
Probiotics	5	500	31	469	27	4	0	6.23 ± 0.80^b^
Fasted + probiotics	5	500	37	463	32	3	2	7.41 ± 0.93^b^

^*^Number of cells examined per group

^**^Class 0 = no detectable DNA damage and no tail; class 1 = tail with a length less than the diameter of the nucleus; class 2 = tail with a length between 1 × and 2 × the diameter of the nucleus; and class 3 = tail with a length more than 2 × the diameter of the nucleus

Mean values within tissues with different superscript letters (^a, b^) are significantly different (P < 0.05)

### Expression of ROS-Related Genes

**Fig. 1:**
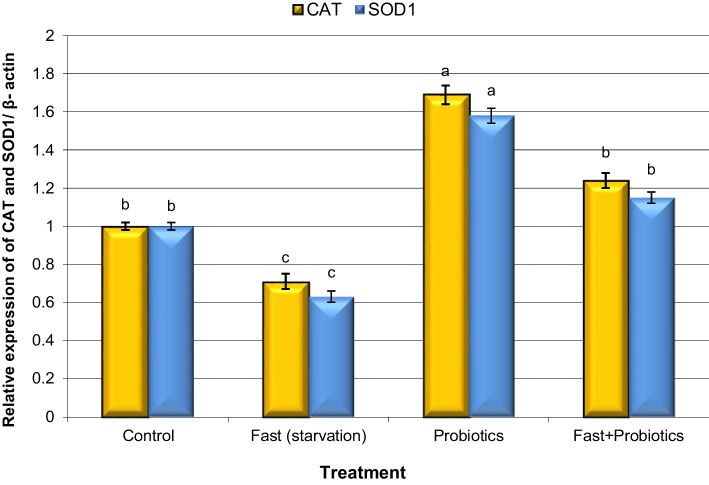
Alteration of the expression of catalase (CAT) and superoxide dismutase 1 (SOD1) genes in liver tissue samples of control and fasted rabbits fed a standard diet with probiotics. Means with different superscripts (^a, b, c^) between groups in the same treatment week are significantly different at P < 0.05. Data are presented as mean ± SEM

**Fig. 2:**
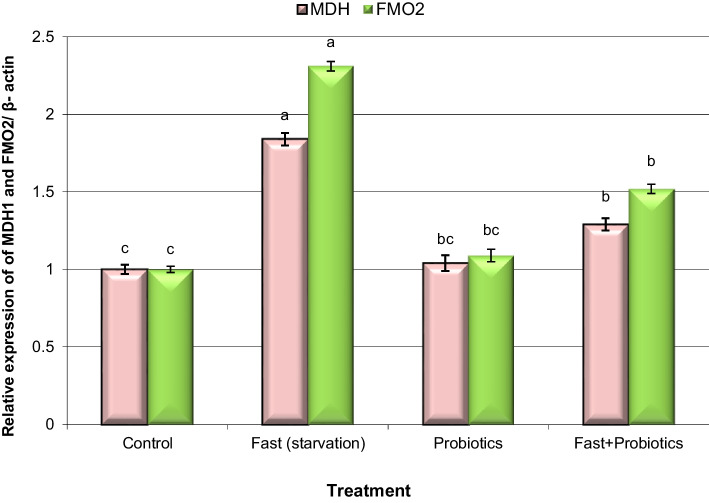
Alteration of the expression of MDH1 and FMO2 genes in liver tissue samples of control and fasted rabbits fed a standard diet with probiotics. Means with different superscripts (^a, b, c^) between groups in the same treatment week are significantly different at P < 0.05. Data are presented as mean ± SEM

Analysis of the expression of genes encoding antioxidant enzymes (CAT and SOD1) and ROS-producing genes (MDH1 and FMO2) in liver samples is illustrated in Figs. 1 and 2.

The expression levels of CAT and SOD1 genes were significantly (P < 0.05) decreased in fatsted rabbits compared with those in control rabbits (Fig. 1). Conversely, the expression levels of CAT and SOD1 genes raised significantly (P < 0.05) in both fasted groups, and fasted rabbits fed a standard diet with the probiotic group compared with those in fasted rabbits.

On the other hand, the expression levels of MDH1 and FMO2 genes were significantly (P < 0.05) up-regulated in fasted rabbits compared with those in control rabbits (Fig. 2). In contrast, the expression levels of MDH1 and FMO2 genes decreased significantly (P < 0.05) in both of fasted group and fasted rabbits fed a standard diet with probiotic group compared with those in fasted rabbits. These results are in agreement with previous studies that documented that probiotics possess antioxidant and nephroprotective effects through elevation of the expression and activity of antioxidant enzymes [45–48].

### Assessment of Apoptosis

**Fig. 3:**
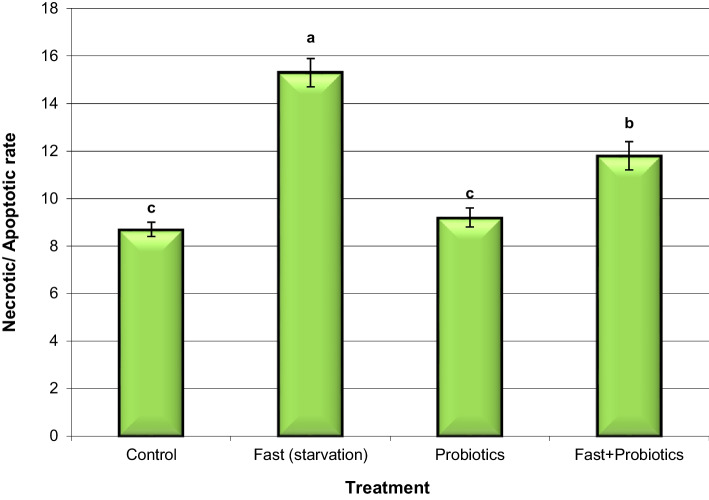
Necrotic/apoptotic rates in liver cell samples from control and fasted rabbits fed standard diet with probiotics. Means with different superscripts (^a, b, c^) between groups in the same treatment week are significantly different at P < 0.05. Data are presented as mean ± SEM

Figure 3 shows the effect of probiotics against oxidative stress-induced apoptosis in fasted rabbits. The rates of necrosis and apoptosis in liver cells of fasted rabbits were increased considerably by 175.9% (15.3 ± 0.61) compared with those in control rabbits (8.7 ± 0.34). Nevertheless, the rates of necrosis and apoptosis in liver cells of rabbits supplemented with probiotics were close (9.2 ± 0.42) to those in control rabbits (8.7 ± 0.34). Additionally, fasted rabbits supplemented with probiotics exhibited a significant (P < 0.05) decline in the rates of necrosis and apoptosis (11.8 ± 0.67) in comparison with those in fasted rabbits (15.3 ± 0.61). Tiptiri-Kourpeti et al. [49] documented that probiotics exert antiproliferative effects. In addition, Blythe et al. [50] reported that intermittent fasting and consumption of probiotic yogurt are associated with immune modulation, detoxification, and antiproliferative effects.

## Conclusion

This study showed that supplementation with probiotics accompanied by a fasting program significantly improved the antioxidant status of postweaning male White New Zealand rabbits by elevating the antioxidant indexes, such as MDA level and CAT, SOD, GSH-Px, and GST activities. Further, this improvement in serum antioxidant indexes seemed to be more effective than in the probiotics supplementation group. In addition, probiotics accompanied by a fasting program decreased the expression of ROS-related genes. Moreover, they could improve hormonal status in postweaning rabbits by reducing T3 and cortisol levels and increasing the level of glucose. This study suggests that supplementation with probiotics accompanied by a fasting program could be used in the future to alleviate oxidative stress, ROS, mortality, and morbidity, thereby contributing to the protective postweaning of rabbits.

## Data Availability

All data and materials are available.
